# Using Conversation Analysis to Examine When Educational Videos Are Introduced in Patient Education

**DOI:** 10.1093/geroni/igab046.091

**Published:** 2021-12-17

**Authors:** Sean Halpin, Michael Konomos, Kathryn Roulston

**Affiliations:** 1 University of Georgia, Decatur, Georgia, United States; 2 Emory University, Atlanta, Georgia, United States; 3 University of Georgia, University of Georgia, Georgia, United States

## Abstract

As the seas of advanced therapies have swelled in the last few decades, multiple myeloma patients have been empowered, encouraged, and sometimes required, to engage in their care. We applied a conversation analysis approach to 12 nurse-led education visits (1011 minutes of audio) containing reference to educational videos. We indexed extracts based on whether the nurse or patient first mention the video. Patients oriented toward the video to demonstrate knowledge (n=15; 88%) and clarify information (n=2; 12%). Nurses oriented toward the video either through positive (n=14; 39%), negative (n=13= 36%), or neutral (n=9; 25%) assessments. Videos created opportunities for patients to pursue their topics of interest during in-person visits. Also, nurses often oriented toward the videos pre-emptively to achieve their teaching goals either by acknowledging the patients’ exposure to upcoming information or by positively or negatively assessing the videos in ways that enable them to re-orient talk to educational scripts.

